# Roles of hypoxia-inducible factor in hepatocellular carcinoma under local ablation therapies

**DOI:** 10.3389/fphar.2023.1086813

**Published:** 2023-02-06

**Authors:** Chunying Xiao, Sheng Liu, Ge Ge, Hao Jiang, Liezhi Wang, Qi Chen, Chong Jin, Jinggang Mo, Jin Li, Kunpeng Wang, Qianqian Zhang, Jianyu Zhou

**Affiliations:** ^1^ Department of Ultrasound, Taizhou Central Hospital (Taizhou University, Hospital), Taizhou, Zhejiang, China; ^2^ Department of Hepatobiliary Surgery, Hubei Cancer Hospital, Tongji Medical College, Huazhong University of Science and Technology, Wuhan, China; ^3^ Department of General Surgery, Taizhou Central Hospital (Taizhou University, Hospital), Taizhou, Zhejiang, China; ^4^ Precision Medicine Center, Taizhou Central Hospital (Taizhou University Hospital), Taizhou, Zhejiang, China; ^5^ Liyuan Hospital, Tongji Medical College, Huazhong University of Science and Technology, Wuhan, China

**Keywords:** hepatocellular carcinoma, hypoxia-inducible factor, ablation, therapy, mechanism

## Abstract

Hepatocellular carcinoma (HCC) is one of the most common digestive malignancies. HCC It ranges as the fifth most common cause of cancer mortality worldwide. While The prognosis of metastatic or advanced HCC is still quite poor. Recently, locoregional treatment, especially local ablation therapies, plays an important role in the treatment of HCC. Radiofrequency ablation (RFA) and high-intensity focused ultrasound (HIFU) ablation are the most common-used methods effective and feasible for treating HCC. However, the molecular mechanisms underlying the actions of ablation in the treatments for HCC and the HCC recurrence after ablation still are poorly understood. Hypoxia-inducible factor (HIF), the key gene switch for adaptive responses to hypoxia, has been found to play an essential role in the rapid aggressive recurrence of HCC after ablation treatment. In this review, we summarized the current evidence of the roles of HIF in the treatment of HCC with ablation. Fifteen relevant studies were included and further analyzed. Among them, three clinical studies suggested that HIF-1α might serve as a crucial role in the RAF treatment of HCC or the local recurrence of HCC after RFA. The remainder included experimental studies demonstrated that HIF-1, 2α might target the different molecules (e.g., BNIP3, CA-IX, and arginase-1) and signaling cascades (e.g., VEGFA/EphA2 pathway), constituting a complex network that promoted HCC invasion and metastasis after ablation. Currently, the inhibitors of HIF have been developed, providing important proof of targeting HIF for the prevention of HCC recurrence after IRFA and HIFU ablation. Further confirmation by prospective clinical and in-depth experimental studies is still warranted to illustrate the effects of HIF in HCC recurrence followed ablation treatment in the future.

## Introduction

Hepatocellular carcinoma (HCC) is the third most common cancer and the fifth most common cause of cancer mortality worldwide (Calderaro et al., 2022; Sperandio et al., 2022). The high mortality of HCC is attributed to the lack of early detection and few effective therapies, especially for intermediate- or advanced HCC patients (Parikh and Pillai, 2021). With the development of medical technology, various new treatment techniques have brought new opportunities for HCC treatment. At present, hepatectomy, liver transplantation, and ablation are the standard treatment for HCC (Zhuang et al., 2021). However, the advanced neoplastic stage and the shortage of donors limit the application of hepatectomy and liver transplantation. Recently, locoregional treatment, especially local ablation therapies, plays an important role in the treatment of HCC.

Local thermal ablation techniques mainly include radiofrequency ablation (RFA), high-intensity focused ultrasound (HIFU) ablation, laser ablation, and microwave ablation (Jing et al., 2020). Among these, RFA and HIFU have been most frequently used worldwide in the treatment of HCC. RFA heats targeted tissue by ionic friction from current, reducing the local increase in temperature above 60 °C and then causing coagulative necrosis (Yousaf et al., 2020). HIFU employs the ultrasonic wave to heat tumor entities, resulting in coagulative necrosis of tumor tissue (Sofuni et al., 2022). Local ablation therapy is more secure and has fewer complications and shorter hospital stays than hepatectomy (Shin et al., 2021). In addition, RFA can be combined with other therapies to treat HCC, thereby providing a better therapeutic effect and overcoming its limitations. For example, a randomized, controlled pilot study reported that RFA combined with TACE showed better effectiveness than RFA alone for HCC (Morimoto et al., 2010). Nevertheless, due to residual viable tumors after local ablation, the rate of recurrence and metastasis is higher than that for surgical resection in HCC. It is reported that the 5-year overall recurrence rates were 63.5% with RFA, while surgical resection was 41.7% in patients with HCC(Huang et al., 2010). As a result, further studies of the molecular mechanism of HCC relapses after local ablation are thus needed so that novel medication targets can be developed.

Local thermal ablation damage has been found to be divided into three regions (central high-temperature zone, sublethal temperature transition zone, and surrounding normal tissue) (Sauvaget et al., 2022). In the transition zone, the damage of tumors can be reversed and eventually survive, thus resulting in the rapid development of tumors. There are increasing studies suggesting that epithelial-mesenchymal transitions (EMT), autophagy, and the hypoxic microenvironment play crucial roles in subsequent progression and metastasis after local ablation. Li et al. (2022) found that insufficient RFA (IRFA) promoted proliferation, invasion, migration, and EMT in HCC cells. Zhao et al. (2018) reported that autophagy has been shown to be activated in mice exposed to IRFA. Importantly, hydroxychloroquine (HCQ), a well-established inhibitor of autophagy, significantly suppressed HCC proliferation and recurrence induced by IRFA (Zhao et al., 2018). Recently, Frenzel et al. (2018) suggested that hypoxia-inducible factor (HIF), the key gene switch for adaptive responses to hypoxia, played an important role in the rapid aggressive recurrence of HCC after RFA. Currently, there is still a lack of narrative reviews on the role of HIF in the local ablation of HCC. In this review, we present the first attempt to comprehensively summarize the recent advances of HIF in local ablation of HCC. The aim of this article is to facilitate the clinical application of HIF in inhibiting the rapid aggressive recurrence of HCC after RFA.

## Overview OF HIF

HIF, a heterodimeric transcription factor, plays a pivotal role in the ability to adapt to changes in oxygen levels (Kling et al., 2021). HIF was first described by Semenza and others in 1995 (Wang et al., 1995; Wang and Semenza, 1995). In 1991, he found a hypoxic inducible nuclear factor, bounding to the promoter of the EPO gene, and then increasing its expression (Semenza et al., 1991). This nuclear factor has been named HIF by Semenza and others in 1995 (Wang et al., 1995; Wang and Semenza, 1995). The huge importance of this discovery was reflected by the 2019 award of the Nobel Prize in Physiology or Medicine (Fitzpatrick, 2019). HIF is composed of α and β subunits. The α subunit is modulated in an oxygen-dependent manner but the β subunit is constitutively expressed (Macedo-Silva et al., 2020). There are three variants of the α subunit, i.e., HIF-1α, HIF-2α, and HIF-3α, and three paralogues of the β subunit, i.e., HIF-1β, HIF-2β, and HIF-3β (Tsai et al., 2020). HIF-1α is the most ubiquitously expressed of the three isoforms and has 48% amino acid sequence identity to HIF-2α (Fitzpatrick, 2019). It has been reported that HIF-1α and HIF-2α played a key role in the acute and chronic response to hypoxia, respectively (Galan-Cobo et al., 2015; Guo et al., 2021). Due to the existence of multiple HIF-3α variants, it is less well characterized. HIF-α is regulated by oxygen-dependent pathways. The HIF-α subunits are synthesized at a very high rate but undergo rapid degradation *via* oxygen-dependent prolyl hydroxylase (PHD) enzymes in the presence of oxygen (Strowitzki et al., 2019). The PHD enzymes could hydroxylate Pro402 and Pro564, two conserved proline residues (Snell et al., 2014). The post-translational hydroxylation enables the von Hipple-Lindau (VHL) to bind the HIF-α subunit for degradation (Liu et al., 2018). The human genome encodes three types of PHD enzymes, i.e., PHD1, 2, and 3 (Fujita et al., 2012). It has been reported that PHD2 regulated HIF-1α, while PHD1 regulated HIF-2α(Ren et al., 2011; Vara-Perez et al., 2021). As oxygen availability decrease, the PHDs activity is diminished, leading to the translocation of HIF-α into the nucleus (Nguyen et al., 2021). HIF-α binds to a hypoxia response element in the nucleus and results in the activation of target genes, which facilitates adaptation and survival of cells and contributes to angiogenesis, proliferation, and metastasis of the tumor (Kaelin and Ratcliffe, 2008).

Mounting evidence demonstrates that HIF may correlate with numerous human diseases (e.g., breast cancer, cervix cancer, and HCC) (Feng et al., 2020; Lal et al., 2021). As known, hypoxia is a feature of most solid tumors, where the oxygen level is usually below 1% (D'Ignazio et al., 2017). A series of reactions is caused by hypoxia, which affects tumor survival and progression and confers resistance to chemoradiotherapy by influencing angiogenesis and metabolism (Muz et al., 2015). HIF has been shown to be the main regulator of these responses (Rocha, 2007). Previous studies have shown that the expression levels of HIF-1α and/or HIF-2α were increased in various tumors such as breast cancer, prostate cancer, and pancreatic cancer, and were correlated with poor survival (Shaida et al., 2008; Zhang et al., 2017; De Francesco et al., 2018; Zhang H. S et al., 2019). Also (Hu et al., 2021), demonstrated that hypoxia significantly induced high expression of HIF-1α and HIF-2α, which promoted the proliferation, migration, and invasion of HCC cells. It was reported that the enhancement of HIF activity promoted tumor metastasis through the regulation of hundreds of genes related to immune escape, cancer stem cell maintenance, angiogenesis, and EMT (Rankin and Giaccia, 2016; Schito and Semenza, 2016). In addition to regulation by PHD and VHL mentioned above, the expression of HIF is also regulated by inflammation and epigenetic regulator in HCC. In the presence of persistent hypoxia, IL-1β is released by the necrotic debris of HCC cells, which promotes HIF-1α synthesis *via* cyclooxygenase 2 (COX-2) (Zhang et al., 2018). The histone deacetylase 5 (HDAC5), an epigenetic regulator, is essential for activating gene transcription and is also involved in the development and progression of tumors (Zhou et al., 2021a). The expression of HDAC5 has been suggested to be elevated in HCC tissues and cells compared with non-cancerous HCC tissues and control cells (Feng et al., 2014). The elevated expression of HDAC5 can induce the transcription of HIF-1α and lead to high expression of HIF-1α by silencing homeodomain-interacting protein kinase-2 (HIPK2) (Ye et al., 2017).

Studies have shown a close association between HIF and recurrence after ablation (Ikemoto et al., 2017). Previous studies indicated that ablation caused hypoxia adjacent to the ablated area, which is related to tumor proliferation, angiogenesis, and aggressiveness (Wan et al., 2016a; Gong et al., 2019). Moreover, the hypoxic status induced by ablation elicits HIF accumulation (Zhang J et al., 2021). (Wan et al., 2016b) discovered that the HSP70/HIF-1α signaling pathway was significantly enhanced in hyperthermia-induced lung cancer cells. Meanwhile, hyperthermia-induced lung cancer cells possess higher proliferation and angiogenesis potential (Wan et al., 2016a). The analogous result was observed in HCC. Kong et al. (2012) found that the expression level of HIF-1α was upregulated in heat-treated HCC cells. Also, a stronger pro-angiogenic effect was observed in heat-treated HCC cells (Kong et al., 2012). Importantly, this effect could be inhibited by HIF-1a inhibitor YC-1 (Kong et al., 2012). These results suggested that HIF may play a crucial role in the rapid progression of residual cancer after ablation.

### Literature search

To maximally identify the eligible articles related to the role of HIF in HCC after ablation, we carried out a systematic search of MEDLINE, Google Scholar, EMBASE, and Cochrane Library databases. The search keyword in MEDLINE was: ((((((((((((((((((((“Carcinoma, Hepatocellular" [Mesh]) OR (Carcinomas, Hepatocellular)) OR (Hepatocellular Carcinomas)) OR (Liver Cell Carcinoma, Adult)) OR (Liver Cancer, Adult)) OR (Adult Liver Cancer)) OR (Adult Liver Cancers)) OR (Cancer, Adult Liver)) OR (Cancers, Adult Liver)) OR (Liver Cancers, Adult)) OR (Liver Cell Carcinoma)) OR (Carcinoma, Liver Cell)) OR (Carcinomas, Liver Cell)) OR (Cell Carcinoma, Liver)) OR (Cell Carcinomas, Liver)) OR (Liver Cell Carcinomas)) OR (Hepatocellular Carcinoma)) OR (Hepatoma)) OR (Hepatomas)) AND (((Hypoxia Inducible Factor) OR (Hypoxia-Inducible Factor)) OR (HIF))) AND (Ablation). The inclusion criteria are as follows: 1) clinical study reporting on the roles of HIF in HCC after ablation; 2) experimental research reporting on the effects of HIF in HCC after ablation and its possible molecular mechanisms. Finally, fifteen clinical or experimental studies were included for further analysis. The researchers then used a specific data collection table to extract relevant data from each study, including the general information of the article (e.g., the first author’s name, the year of publication), research subject (e.g., cell/animal model or patient), ablation surgical type, associated genes/pathways and agents, measure method of HIF, and the major findings of the study. Table 1 summarizes the relevant studies reporting the roles of HIF on HCC after ablation.

**TABLE 1:** The characteristics and the main findings of the included studies.

Study/Reference	Research subject	Ablation surgical type	Measure method	Associated genes/pathways and agents	Main findings
Kong et al. (2012)	HCC cells	RFA	Western blot	Up-regulated	RFA promoted the growth of residual HCC by inducing angiogenesis *via* HIF-1α/VEGFA pathway
				HIF-1α/VEGFA pathway	
Halpern et al. (2021)	Mice	RFA	Western blot and PCR	Up-regulated HIF-1α/arginase-1 or HIF-1α/VEGF pathway	RFA led to an increase in engraftment and progression of hepatic metastases by enhancing HIF-1α expression
Chen et al. (2021)	Nude mice	Microwave ablation	Western blot and PCR	Enhanced the Warburg effect	HIF-1α promoted HCC progression after thermal ablation by enhancing the Warburg effect
Wu et al. (2014)	Nude mice	HIFU ablation	Immunohistochemistry, Western blot and PCR	Enhanced the HIF-1, 2α/VEGFA/EphA2 pathway	HIFU ablation enhanced pro-angiogenic effect by HIF-1, 2α/VEGFA/EphA2 pathway in the residual hepatocellular carcinoma
Wu et al. (2017)	Nude mice	HIFU ablation	Immunohistochemistry and Western blot	Enhanced the HIF-2α/VEGFA/EphA2 pathway	HIFU ablation induced residual tumor angiogenesis by up-regulating HIF-2α/VEGFA/EphA2 pathway in HCC.
Xu et al. (2013)	Nude mice	RFA	PCR and immunohistochemistry	Up-regulation of HIF-1α/VEGFA pathway	Insufficient RFA promoted recurrence of HCC by increasing HIF-1α and VEGFA expression
Nijkamp et al. (2009)	Male BALB/c mice and male Wag/Rij rats	RFA	Western blot and immunohistochemistry	Up-regulation of HIF-1, 2α and downstream markers CA IX and VEGF	RFA induced the outgrowth of tumor cells through the up-regulation of HIF-1, 2α pathways in animal model of colon cancer hepatic metastasis
Tong et al. (2017)	HCC cells	RFA	Western blot	HIF-1α induced EMT	HIF-1α increased the migration, invasion, and sorafenib chemoresistance by inducing EMT after RFA.
Xu et al. (2019)	HCC cells	RFA	Western blot	Up-regulation of HIF-1α/BNIP3 pathway	IRFA promoted residual HCC cell progression by enhancing autophagy *via* up-regulation of HIF-1α/BNIP3 pathway
Wu et al. (2014)	BALB/c nu/nu mice	HIFU ablation	Immunohistochemistry, Western blot and PCR	Up-regulation of HIF-2α/VEGF pathway	HIFU treatment promoted angiogenesis by up-regulating HIF-2α/VEGFA pathway in mice with hepatocellular carcinoma
Wu et al. (2021)	Nude mice	HIFU ablation	Western blot, PCR and immunohistochemistry	Sorafenib inhibited the HIF-2α/VEGF-A/EphA2 pathway	Sorafenib inhibited HIFU ablation-induced progression of the residual tumor by suppressing HIF-2α/VEGF-A/EphA2 pathway in HCC.
Yamada et al. (2014)	Patients (n = 88)	RFA	PCR	Up-regulation of HIF-1α and EpCAM	The RFA group showed aggressive tumor phenotype and poor prognosis by enhancing HIF-1α and EpCAM expression in the residual HCC tumors
Yuan et al. (2017)	Patients (n = 144)	RFA	ELISA	TACE inhibited HIF-1α and EGR2	TACE combined with RFA reduced tumor cell proliferation speed by inhibiting the expression of HIF-1α
Dong et al. (2022)	HCC cells and nude mice	RFA	Western blot and immunohistochemistry	ATO inhibited p-Akt/HIF-1α pathway	ATO inhibited angiogenesis in HCC by blocking Ang-1 and Ang-2 through the inhibition of p-Akt/HIF-1α pathway
Gong et al. (2017)	Patients (n = 90)	RFA	ELISA	Sorafenib reduced VEGF, CTGF, HIF-1α and OPN expression	Sorafenib combined with RFA showed a superior overall treatment efficacy than only RFA by inhibiting the expression of VEGF, CTGF, HIF-1α and OPN in HCC.

Note: HCC = Hepatocellular carcinoma; RFA = Radiofrequency ablation; HIF-1α = Hypoxia inducible factor-1 alpha; VEGF = Vascular endothelial growth factor; VEGFA = Vascular endothelial growth factor A; HIFU = High-intensity focused ultrasound; HIF-2α = Hypoxia inducible factor-2 alpha; EphA2 = Epithelial cell kinase; CA IX = Carbonic anhydrase IX; EMT = Epithelial-mesenchymal transition; BNIP3 = Bcl-2/adenovirus E1B 19 kDa interacting protein 3; EpCAM = Epithelial cell adhesion molecule; TACE = Transcatheter arterial chemoembolization; EGR2 = Early growth response protein 2; ATO = Arsenic trioxide; CTGF = Connective tissue growth factor; OPN = Osteopontin.

## Clinical implications of HIF-1α in HCC after ablation

Among the 15 included studies, three clinical studies reported the clinical roles of HIF-1α on HCC after ablation. Yamada et al. (2014) conducted a clinical study of Eighty-eight (88) patients with HCC. These patients were categorized into two groups (i.e., RFA group and non-RFA group). There was no significant difference in TMN staging, number of tumors, maximum tumor diameter, and tumor markers between the two groups. The overall 5-year survival rate of the RFA groups was lower than those of non-RFA groups (39% vs. 68%; *p* = 003). As identified through qRT-PCR, the RFA group showed increased expression not only of HIF-1α, but also of epithelial cell adhesion molecule (EpCAM), a marker for tumor cell growth and metastasis, compared to the non-RFA group. Of note, the expression level of angiogenic factors VEGF was not different between the two groups. Yamada et al.‘s study revealed that the local recurrence of HCC after RFA was significantly associated with the increased expressions of HIF-1 and EpCAM, but not the expressions of VEGF (Yamada et al., 2014). As is well known, sorafenib, an oral multi-kinase inhibitor, can block the proliferation and induce cell apoptosis, which has been widely to treat advanced HCC. Gong et al. (2017) assigned 50 patients with HCC to control group and 40 patients with HCC to observation group. Two groups of patients were treated with conventional RFA alone and with a combination of RFA and oral sorafenib, respectively (Gong et al., 2017). The patients in the observation group showed more prolonged tumor-free survival (12.3 vs. 8.4 months), lower relapse (15.0% vs. 34.0%), and longer survival rates (87.5% vs. 70.0%) than those in the control group (Gong et al., 2017). Furthermore, the overall treatment efficacy in the observation group was superior to that in the control group (82.5% vs. 62.0%); however, the incidence of complications showed no differences between the two groups (17.5% vs. 20.0%) (Gong et al., 2017). The author further found that in comparison to the average levels before treatment, the average levels of serum VEGF and HIF-1α were decreased after the treatment (Gong et al., 2017). This study suggested that sorafenib in combination with RFA was a superior treatment for HCC and HIF-1α and VEGF played key roles during the treatment process. Similarly, another study performed by Yuan et al. (2017) showed that the combination of transcatheter arterial chemoembolization (TACE) and RAF significantly decreased liver cancer angiogenesis and reduced HCC cell proliferation speed than TACE alone. Moreover, the TACE combined with RAF reduced the expression levels of HIF-1α and early growth response protein 2 (EGR-2) to a greater extent than TACE alone (Yuan et al., 2017). In summary, the above three clinical studies suggested that HIF-1α might serve a crucial role in the RAF treatment of HCC or the local recurrence of HCC after RFA.

## The roles of HIF and the related signaling pathway in HCC after ablation

### HIF-1α contributes to the progression of HCC by promoting the expression of VEGFA and arginase-1

Arginase, a pivotal metabolic enzyme, is a part of the urea cycle and catalyzes the hydrolysis of L-arginine, resulting in ammonia detoxification in mammals (Cziraki et al., 2020). It has two isoforms: arginase-1 and arginase-2, localized in the cytosol and mitochondria, respectively (Labib et al., 2020). Arginase-1 is primarily detected in hepatocytes and can be served as an important marker of hepatocellular differentiation (Moudi et al., 2020). Recently, arginase-1 has been reported to be induced in activated macrophages and participates in the initiation and progression of various diseases, including HCC (You et al., 2018). In addition, HIF-1α is reported to be involved in the expression of the arginase-1 (Alexander et al., 2020). The HIF well-studied target is the vascular endothelial growth factor A (VEGFA) (Palazon et al., 2017). It is the main stimulator of tumor angiogenesis and is reported to be overexpressed in multiple solid cancers, including HCC (Zou et al., 2020). Lin et al. (2018) demonstrated that long non-coding RNA UBE2CP3, a cancer-promoting gene of HCC, enhanced the progression of liver cancer by promoting angiogenesis through the activation of ERK1/2/HIF-1α/VEGFA signaling pathway. A recent study conducted by Halpern et al. (2021) showed that a metastatic tumor model was established using a splenic injection of colon adenocarcinoma cells, and all mice undergoing RFA developed tumors at the ablation site on necropsy performed at 7 days. In contrast, the site of probe insertion had no tumors in the mice undergoing a sham procedure (Halpern et al., 2021). Furthermore, the expression of HIF-1α and VEGFA was significantly increased at the ablation site relative to unaffected adjacent liver tissues from the same mouse (Halpern et al., 2021). The expression of mRNA of HIF-1α, VEGFA, and arginase-1 were also significantly elevated in RFA/tumor-associated macrophages compared with that in unaffected liver (Halpern et al., 2021). Further study found that the tumor volume and the median number of total metastases were significantly decreased in mice treated with YC-1, a well-established inhibitor of HIF-1α, compared to vehicle control (Halpern et al., 2021). In agreement with the above study, Xu et al. (2013) found that the expression of HIF-1α and VEGFA were significantly increased in hepatic tumor model mice after RFA and this response could be inhibited by the combination of sorafenib and RFA. Therefore, HIF-1α might promote the development of hepatic metastases after RFA by increasing the expression of VEGFA and arginase-1.

HIF-1 and 2α promote angiogenesis and residual recurrence of HCC after HIFU ablation *via* VEGFA/EphA2 pathway.

High-intensity focused ultrasound (HIFU) is another local ablative therapy and is also difficult to achieve complete ablation of HCC, which contributes to the recurrence of HCC. Erythropoietin-producing hepatocellular A2 (EphA2), a member of the Eph family of receptor tyrosine kinases, is a crucial regulator of tumorigenesis and is highly expressed in multiple cancers, including HCC (Wilson et al., 2021). EphA2 has been shown to be activated through the phosphorylation on serine 897 mediated by AKT, RSK, and PKA kinases and alters downstream signaling, and then facilitating tumor progression (Zhou et al., 2015; Barquilla et al., 2016). Niu et al. (2021) reported that EphA2 was highly expressed in HCC. Additionally, EphA2 silencing significantly reduced cell proliferation and accelerated apoptosis (Niu et al., 2021). A study conducted by Cheng et al. (2002) demonstrated that EphA2 antisense oligonucleotides significantly suppressed endothelial expression of the EphA2 receptor and inhibited VEGF-induced cell migration. A recent study showed that the knockdown of HIF-1α by siRNA downregulated the expression of EphA2 and led to apoptosis and the disruption of vasculogenic mimicry (VM) associated phenotypes (Saha et al., 2022). Nevertheless, it is unknown whether EphA2 functions in HCC after HIFU ablation. Wu et al. (2014) investigated the roles of EphA2, VEGFA, and HIF-1α in a xenograft model of HCC in nude mice. The authors found that the protein and mRNA levels of HIF-1α and HIF-2α were significantly increased in the residual tumor tissues of the HIFU group compared to that in the control group (Wu et al., 2014). Furthermore, similar results were obtained for VEGF-A and EphA2 expression (Wu et al., 2014). The expression of CD31 is considered an indicator of calculated the microvascular density (MVD) and is significantly increased compared with the control grou p (Wu et al., 2014). Importantly, the alteration of CD31, VEGF-A, and EphA2 expression were basically consistent with the trends in HIF-1α and HIF-2α expression (Wu et al., 2014). Therefore, the authors suggested HIFU ablation might result in the hypoxia condition of residual tumor and induce tumor angiogenesis *via* HIF-1, 2α/VEGFA/EphA2 (Wu et al., 2014). Two other studies conducted by Wu et al. (2017); Wu et al. (2021) also demonstrated that overexpression of EphA2, VEGFA, and HIF-2α were closely associated with angiogenesis in residual HCC after HIFU ablation. Moreover, the expression of EphA2, VEGFA, and HIF-2α could be significantly inhibited by sorafenib (Wu et al., 2021). Thus, inhibiting HIF-1, 2α alone or in combination with angiogenesis inhibitors might prevent residual recurrence of HCC after HIFU ablation.

## HIF-1α and HIF-2α promote liver metastases of colon carcinoma following RFA *via* the activation of downstream markers CA-IX and VEGF

The induction of angiogenesis is an important biological process in responseto hypoxia (Ben et al., 2022). Also, active pH regulation is an essential biological process in response to hypoxia, which allows tumor cells to maintain viability and proliferation in the acidic tumor microenvironment (Porter and Porter, 2018). Carbonic anhydrase IX (CA IX) has been proven to be a key component of this pH regulatory machinery (Benej et al., 2020). CA IX, a transmembrane protein, catalyzes the reversible dehydration of bicarbonate. In recent studies, CA IX is served as a potent biomarker of poor patient prognosis for many types of solid tumors, including HCC (Parks and Pouyssegur, 2017; Chen et al., 2018). Cho et al. (Cho et al., 2019) demonstrated that CA IX has overexpressed in HCC and CA IX inhibitor (acetazolamide) significantly suppressed the growth of HCC xenograft tumors in nude mice. In addition, HCC patients with high CA IX expression displayed a worse prognosis in the TCGA database (Cho et al., 2019). CA IX has been reported to be a downstream marker of HIF (Nijkamp et al., 2009). Nijkamp et al. (2009) established preclinical models with colorectal micrometastases to investigate the effect of RFA on the outgrowth of tumor cells at the lesion periphery. The authors found that tumor load in the transition zone (TZ) significantly increased after RFA compared with tumor load in the livers of sham-operated mice (48.5 ± 3.9% vs. 17.9 ± 2.0%, *p* = 0.00021) (Nijkamp et al., 2009). Furthermore, tumor load in the TZ following RFA had increased approximately 4-fold compared with tumor load in the reference zone (RZ) (Nijkamp et al., 2009). HIF-1α, HIF-2α, CA-IX, and VEGF were significantly up-regulated in the TZ (Nijkamp et al., 2009). As is noted, 17DMAG, an inhibitor of HIF, suppressed the expression of HIF-1α and HIF-2α, and significantly reduced tumor growth in the TZ (Nijkamp et al., 2009). However, 17DMAG had no obvious effect on tumor growth in the RZ of RFA-treated mice (Nijkamp et al., 2009). The above studies suggested that HIF-1, 2α might promote liver metastases of colon carcinoma following RFA by enhancing the expression of CA-IX and VEGF, the downstream markers of HIF.

## HIF-1α/BNIP3 pathway promotes residual HCC cell progression by enhancing autophagy after IRFA

Autophagy is a highly conserved, intracellular self-protective process, critically required for the degradation of damaged organelles and cytoplasmic material (Verma et al., 2021). There are accumulating data showing that autophagy plays a key role in a variety of tumors, including HCC. In pancreatic cancer, autophagy promoted tumor growth in syngeneic host mice, but the inhibition of autophagy reduced tumor growth by restoring surface levels of major histocompatibility complex class I (Yamamoto et al., 2020). In HCC, the expression of LC3-II, a key autophagic marker, was significantly increased and associated with poor prognosis of HCC. Moreover, inhibition of autophagy reduced the ability of tumor cells to survive (Han et al., 2021; Zhang K et al., 2021). Wang et al. (2018) found that the activity of autophagy was markedly enhanced in the residual HCC cells after RFA and the autophagy inhibitor 3-methyladenine (3-MA) significantly inhibited the cell viability and invasion induced by IRFA. Recently, mounting studies have shown that Bcl-2 19-kDa interacting protein 3 (BNIP3) is served as a mitochondrial protein and plays a critical role in autophagy. For example, Xu et al. (2021) reported that latent membrane protein1 (LMP1) promoted radioresistance by inducing autophagy through BNIP3 in nasopharyngeal carcinoma. Additionally, BNIP3 can be induced by hypoxia and is confirmed to be the target molecule of HIF-1α (Chen W et al., 2016). Zhang Y. et al. (2019) demonstrated that HIF-1α played a protective role against myocardial ischemia-reperfusion injury by inducing BNIP3-mediated autophagy. However, the role of HIF-1α/BNIP3-mediated autophagy in RFA-induced HCC promotion remains unclear. A recent study conducted by Xu et al. (2019) showed that the proliferation, migration, and invasion abilities of residual HCC cells were significantly elevated after the IRFA was simulated *in vitro*. Compared with the 37°C-si-NC group, the expression of LC3B-II, HIF-1α and BNIP3 were significantly increased in the 47°C-si-NC group, which indicated that IRFA could induce the activation of autophagy and the upregulation of HIF-1α and BNIP3 in HCC cells (Xu et al., 2019). Notably, BNIP3 silencing significantly decreased the expression levels of HIF-1α and LC3B-II as well as slowed the proliferation, migration, and invasion of HCC cells mediated by IRFA (Xu et al., 2019). These studies revealed that IRFA promoted residual HCC cell progression by inducing autophagy *via* the HIF-1α/BNIP3 pathway.

## ATO inhibits tumor growth and angiogenesis of HCC by blocking the paracrine signaling of Ang-1 and Ang-2 through the inhibition of the p-Akt/HIF-1α pathway after IRFA

Angiogenesis is an essential process in the growth and metastasis of HCC (Zhu et al., 2022). Angiogenesis is not only regulated by VEGF but also by angiopoietin-1 (Ang-1) and angiopoietin-2 (Ang-2), and their receptor Tie-2 (Engin et al., 2012). Ang-1 and Ang-2 are the members of the angiopoietin (Ang) family and regulate angiogenesis *via* the TEK tyrosine kinase endothelial receptor (Lin et al., 2020; Xie et al., 2020). Also, Ang-1 and Ang-2 have been shown to be the prognostic biomarkers of HCC(Lin et al., 2020; Xie et al., 2020). Pestana et al. (2018) revealed that a high level of Ang-2 was closely associated with the poor prognosis of HCC patients. In contrast, HCC patients with a high level of Ang-1 showed a longer overall survival (Pestana et al., 2018). Contrary to the studies discussed above, another study found that activated hepatic stellate cells (aHSCs) promoted angiogenesis through secreting Ang-1 (Lin et al., 2020). Wang Z et al. (2021) reported that morphine significantly promoted angiogenesis by activating Akt/HIF-1α pathway in HCC. However, whether Akt/HIF-1α axis is involved in the progression of residual HCC after RFA by modulating Ang-1 or Ang-2 was still unclear. A more recent study indicated that IRFA was simulated using a water bath and promoted tumor growth and angiogenesis of HCC *in vitro* and *in vivo* (Dong et al., 2022). The above phenomenon could be suppressed by arsenic trioxide (ATO) (Dong et al., 2022). Furthermore, higher levels of Ang-1, Ang-2, and p-Tie2 were detected in a conditioned medium from RFA-treated than from untreated HCC cells (Dong et al., 2022). The expression of HIF-1α and p-Akt were also upregulated following RFA in HCC cells (Dong et al., 2022). The levels of Ang-1, Ang-2, p-Tie2, HIF-1α, and p-Akt were suppressed by ATO (Dong et al., 2022). Further studies found that Ang-1 or Ang-2 knockdown impaired the ability of the conditioned medium to promote angiogenesis (Dong et al., 2022). Similar results can be obtained when Tie2 expression was silenced using siRNA (Dong et al., 2022). In addition, YC-1, an inhibitor of HIF-1α, markedly inhibited the increased expression of Ang-1 and Ang-2 in HCC cells (Dong et al., 2022). The levels of Ang-1, Ang-2, p-Tie2, and p-Akt were upregulated following HIF-1α overexpression, and the effect of ATO was attenuated (Dong et al., 2022). These studies indicated that ATO suppressed tumor growth and angiogenesis of HCC by regulating paracrine Ang-1 and Ang-2 secretion through the p-Akt/HIF-1α pathway after IRFA.

## HIF-1α facilitates the progression of HCC by promoting the warburg effect and EMT after IRFA

Continuous aerobic glycolysis has been demonstrated to trigger oncogene development in the cancer cells. Aerobic glycolysis is known as the Warburg effect and is one of the tumor hallmarks (Liu et al., 2022a). The enhanced Warburg effect is closely linked to EMT and poor patient prognosis in multiple cancer, including HCC. Zhou et al. (2021b) reported that zinc finger E-box-binding homeobox 1 (ZEB1), predicting worse overall survival in cancer patients, facilitated tumorigenesis and metastasis of HCC by enhancing the Warburg effect. It was reported that HIF-1α regulated the Warburg effect by influencing glycolysis, accumulation of lactic acid, and infiltration of the extracellular matrix (Zhou et al., 2022). Lyu et al. (2022) reported that HIF-1α accelerated the Warburg effect and promoted ovarian cancer tumorigenesis by upregulating WT1-associated protein. Liu et al. (2021) demonstrated that by regulating the miR-30c/HIF-1α pathway, FBI-1, an important regulator of HCC, promoted the Warburg effect or EMT of HCC cells and contributed to the drug resistance of HCC cells. A recent study demonstrated that the Warburg effect and level of HIF-1α are enhanced in HCC cells after sublethal heat stress (Chen et al., 2021). 2-NBDG uptake experiment showed that a dramatically increased uptake of glucose was observed in HCC cells after sublethal heat stress (Chen et al., 2021). In addition, HCC cells under normal glucose conditions had a higher survival rates following sublethal heat stress than that under complete glucose-deprivation conditions (Chen et al., 2021). These data indicated that the increased uptake of glucose induced by Warburg effect promoted HCC cell proliferation and invasion (Chen et al., 2021). Further study found that dimethyloxallyl glycine, a specific HIF-1α agonist, improved glucose uptake, and promoted glycolysis-related markers (GLUT-1/3 and LDHA) expression in HCC cells (Chen et al., 2021). Taken together, HIF-1α might facilitate the progression of HCC by regulating the Warburg effect after IRFA.

**FIGURE 1:**
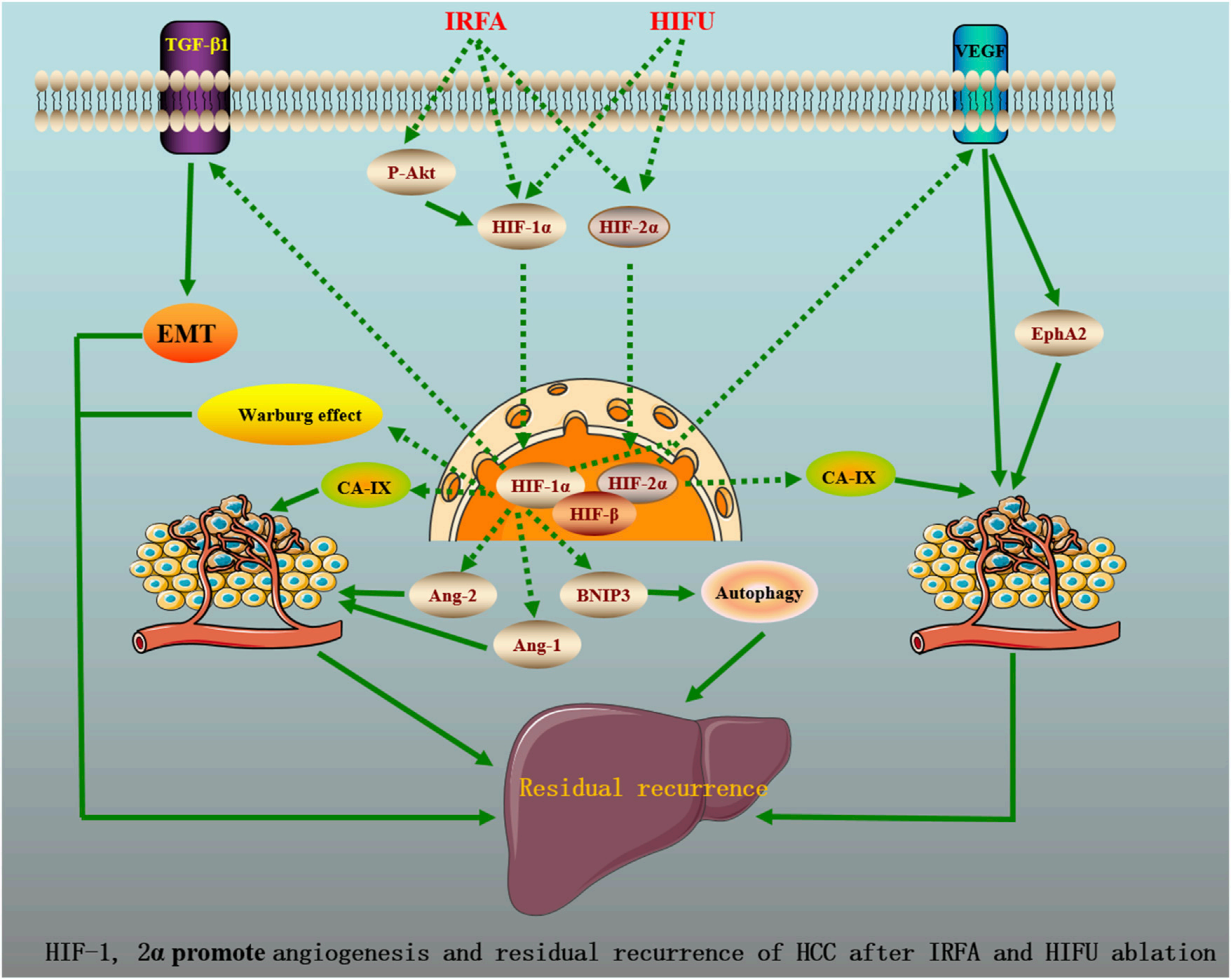
HIF-1, 2a promote angiogenesis and residual recurrence of HCC after IRFA and HIFU ablation.

As known, E-cadherin, N-cadherin, vimentin, and Snail are the markers of EMT (Cessna et al., 2022). Tong et al. (2017) mimicked the IRFA through thermal treatment under hypoxic conditions and found that the protein levels of HIF-1α, N-cadherin, vimentin, Snail, and TGF-β1 following IRFA were significantly elevated, while the expression of E-cadherin was reduced in HCC cells. Moreover, the formation of a hypoxic microenvironment following IRFA significantly promotes HCC cell migration and invasion. Furthermore, N-cadherin, vimentin, Snail, and TGF-β1 expression in shHIF-1α cells were decreased in conjunction with an increased in E-cadherin expression. Previous studies have already demonstrated that TGF-β1 promoted EMT by increasing the expression of vimentin and Snail in HCC (Chen and Yan, 2021; Zhang Z et al., 2021). Of note, upregulation of vimentin and Snail induced by IRFA could be completely inhibited by pretreatment with SB431542, an inhibitor of the TGF-β receptor. All of these data indicated that HIF-1α promoted EMT by TGF-β1 after IRFA, contributing to HCC invasion and metastasis. Figure 1 shows the potential molecular mechanisms of HIF-1, 2α promote angiogenesis and residual recurrence of HCC after IRFA and HIFU ablation.

## Roles of HIF inhibitors for the treatment of HCC and its prospects

von Hippel-Lindau (VHL) serves as a tumor suppressor gene. Hypoxia enhances phagocytosis in neutrophils, and in neutrophils containing a mutated VHL, increased HIF-1α levels lead to decreased apoptosis and increased bacterial phagocytosis under normoxia conditions. HIF-1α can be induced by hypoxia or mutations of VHL. VHL is required to mediate the degradation of HIF-1α in the ubiquitin-proteasome pathway. It was reported that inactivation of the VHL tumor suppressor gene, which results in pseudohypoxia stabilization of both HIF-1α and HIF-2α, is an initiating genetic event for both hereditary (VHL disease) and sporadic renal cell carcinoma (RCC) (Kaelin, 2007). Genetic and functional studies have supported a protumorigenic role of HIF-2α and a tumor suppressor role for HIF-1α in ccRCC, which has prompted the development of HIF-2α–specific inhibitors (Choueiri and Kaelin, 2020). HIF-1α is believed to play an important role during RCC initiation and is elevated in the earliest preneoplastic lesions in VHL patients (Mandriota et al., 2002).

Now, selective HIF-2α antagonists (PT2385 and PT2399) that inhibit HIF-2 transcriptional activity were identified. PT2399 suppressed tumor growth in most human patient-derived xenografts (PDXs) with greater antitumor activity than that of the tyrosine kinase inhibitor (TKI) sunitinib (Chen J et al., 2016). PT2977 (also known as MK-6482, belzutifan), a PT2385 derivative with an improved pharmacokinetic profile and potency, demonstrated promising single-agent activity in heavily pretreated patients with advanced RCC and VHL-associated non-metastatic ccRCC and was recently approved by the FDA for the treatment of cancers associated with VHL disease (Courtney et al., 2020). Inconsistent results were found in the common isoforms of HIF, HIF-1α, and HIF-2α. A recent study using tissue from 380 patients revealed a significant association of high HIF-2α with increased OS, whereas high HIF-1α was significantly associated with higher-grade tumors and reduced OS in both the univariable and multivariable settings (Cowman et al., 2020). As aforementioned, HIF-1α was considered to be a tumor suppressor. This is inconsistent with the finding that high HIF-1α expression was associated with poor survival in RCC (Minardi et al., 2015). Therefore, the inhibition of HIF-1α should continue to be explored as a therapeutic strategy for RCC.

In the HCC setting, HIF-α inhibitors may also play roles in the treatment of HCC. Clinical data have demonstrated that overexpressed HIF-1α and HIF-2α in HCC patients are reliable markers of a poor prognosis (Dai et al., 2018; Mendez-Blanco et al., 2018). At present, only one clinical study was available on ClinicalTrials.gov and Pubmed. This phase I study evaluated the intravenous infusion effect of the HIF- 1α mRNA antagonist RO7070179 in HCC patients failing to respond to systemic therapy, but this study did not show the results [ClinicalTrials.gov Identifier: NCT02564614].

Of note, the majority of studies are preclinical findings. For example, Salman et al. (2022) showed that HIF-1 and HIF-2 inhibitor 32-134D eradicate HCC in combination with anti-PD1 therapy . LW6 is a drug that inhibits hypoxia by reducing HIF-1α accumulation and gene transcriptional activity. Xu et al. (2022) found that LW6 can promote apoptosis of HCC cells by inhibiting HIF-1α, inhibiting tumor angiogenesis, and downregulating the expression of PD-L1, which is an effective choice for the treatment of HCC. Mu et al. (2021) found that HIF-2α knockdown decreased the expression of downstream c-MYC, suppressed hypoxic cell proliferation, and induced HCC cell apoptosis, whereas HIF-1α knockdown did not. Wu et al. the expression of HIF-2α can be inhibited by sorafenib, which is likely to provide an effective adjunct treatment for patients with HCC following HIFU ablation (Wu et al., 2021).

Based on the above evidence, preclinical studies demonstrated that HIF inhibitors have been proposed as one of the effective treatments for HCC. Further clinical investigations would allow for a better understanding and help to propose more effective strategies to increase the efficacy of HIF inhibitors treatment for HCC.

## Combination therapies in the management of early and unresectable HCC

There have been many studies indicative of the therapeutic benefit of the combination of anticancer therapies for early and unresectable HCC, such as RFA combined with a chemotherapeutic drug, and thermal ablation combined with transarterial chemoembolization. The combination of local ablation therapies with immunotherapies represents a promising therapeutic strategy for treating HCC. The primary immunotherapeutic strategies include immune checkpoint inhibitors therapy (e.g., PD-1 and PD-L1), cell-based therapies, and tumor vaccine therapy. Though the role of PD-1 inhibitors in the adjuvant setting is still under investigation, the administration of PD-1 blockade in addition to RFA was found to be promising. A previous study reported that PD-1 inhibitor combined with RFA for recurrent HCC resulted in a significantly improved 1-year RFS rate compared to RFA alone (Wang X et al., 2021). A multicenter RCT (Lee et al., 2015) showed that adjuvant immunotherapy with activated cytokine-induced killer cells increased recurrence-free and OS in patients with HCC who underwent curative treatment with RFA. In addition, Ji et al. (2021) showed that RFA and TACE combined with postoperative autologous cytokine-induced killer (CIK) cell immunotherapy reinfusion have significant efficacy in the treatment of primary HCC. A preclinical study demonstrated that PI3Kγ inhibitors could enhance anti-PD-1 therapy for the treatment of residual tumors after IRFA (Liu et al., 2022b). However, though these studies provide a strong rationale for combining RFA and the immunotherapies in the clinical setting, the roles of HIF in this action are extremely scarce.

Advances are currently being made in this area of HCC with ablation treatment. A single-arm phase 2 trial (Qiao et al., 2022) demonstrated that addition of anti-PD-1 adjuvant therapy after TACE combined with ablation significantly prolong the relapse-free survival with controllable safety for HCC patients with high recurrence risk. Repeat hepatectomy was considered to serve as the first choice for solitary small recurrent HCC patients with late recurrence, while MWA should be selected for those with early recurrence (Wang et al., 2022). A randomized controlled phase 2 trial conducted by Radosevic et al. (2022) showed that MWA created larger ablation zones than RFA (*p* = 0.036). The authors concluded that both MWA and RFA were effectiveness and safety in liver tumors between 1.5 and 4 cm. Suh et al. (2021) revealed that no-touch RFA using twin internally cooled wet electrodes demonstrated significantly lower cumulative local tumor progression rates than conventional RFA for small HCCs. A phase 2 study (Iwata et al., 2021) reported that image-guided proton therapy (IGPT) was a safe and effective treatment for solitary operable or ablation-treatable HCC. Zaitoun et al. (2021) revealed that combined therapy with conventional TACE + MWA is safe, well-tolerated, and more effective than TACE or MWA alone for treatment of HCC with 3–5 cm. Previous study showed that sorafenib combined with TACE and RFA resulted in longer recurrence-free survival and better OS than did TACE-RFA in patients with medium or large HCC (Zhu et al., 2018). However, a subsequent study developed by Bockorny et al. (2022) suggested that priming of sorafenib did not enhance the effect of RFA in intermediate sized HCC. The above studies showed that recent studies continue to explore more effective therapeutic strategies by combining hepatectomy or ablation for treating different staging of HCC (Table 2).

**TABLE 2:** Main findings of the recent clinical trials in the field of HCC ablation.

Study/Reference	Main findings
Qiao et al. (2022)	Addition of anti-PD-1 adjuvant therapy after TACE combined with ablation significantly prolong the relapse-free survival with controllable safety for HCC patients with high recurrence risk
Wang et al. (2022)	Repeat hepatectomy should be the first choice for solitary small recurrent HCC patients with late recurrence, while MWA should be selected for those with early recurrence
Radosevic et al. (2022)	MWA created larger ablation zones than RFA (*p* = 0.036) although without differences in short-to-long diameter ratio of ablation zone. Both MWA and RFA are effectiveness and safety in liver tumors between 1.5 and 4 cm
Suh et al. (2021)	No-touch RFA using twin internally cooled wet electrodes demonstrated significantly lower cumulative local tumor progression rates than conventional RFA for small HCCs
Iwata et al. (2021)	A phase 2 study demonstrated that image-guided proton therapy (IGPT) is a safe and effective treatment for solitary operable or ablation-treatable HCC
Zaitoun et al. (2021)	Combined therapy with conventional TACE + MWA is safe, well-tolerated, and more effective than TACE or MWA alone for treatment of HCC with 3–5 cm
Bockorny et al. (2022)	Priming of sorafenib did not enhance the effect of RFA in intermediate sized HCC.

With the advance of screening technology and increased awareness of cancer surveillance, more and more HCC could be detected at early stage, rendering curative therapeutics applicable. The therapeutic strategies of HCC are evolving rapidly. In the 2022 update of BCLC strategy for HCC management, local ablation still plays leading part among the recommended curative treatments for early-stage HCC (Chen S et al., 2022). As compare to the less effective alcohol injection, an increased use of radiofrequency ablation has been found to improve management of intermediate stage patients with HCC (Garuti et al., 2021). Adjuvant therapies (e.g. immunotherapy) have been found to prevent against HCC recurrence after curative treatment, which could significantly improve the prognosis. For the combination with imunotherapy, ablative techniques are gaining more and more attention for their capability of boosting local and systemic immune effects, which makes combination strategy a promising weapon for HCC treatment (Chen M et al., 2022). Besides, tyrosine kinase inhibitors, oncolytic virotherapy, and cancer vaccines, may also boost anti-tumor immunity for HCC. These novel therapeutic strategies show great potential to synergize with ablation in the treatment of primary and metastatic HCC.

It was reported that sorafenib in combination with RFA could improve the treatment of HCC due to sorafenib suppresses cell proliferation and induces apoptosis in hepatoma cells by the HIF-1/VEGFA pathway (Gong et al., 2017). The combination of current sorafenib treatment with gene therapy or inhibitors against HIFs has been documented as promising approach to overcome sorafenib resistance both *in vitro* and *in vivo*. Wu et al. (2021) reported that the synergistic effect of the combination of HIFU with sorafenib therapy inhibited HCC tumor growth when compared with HIFU treatment alone or with no treatment, which was closely related to the decreased HIF-2α, VEGFA. Gong et al. (2017) also found that sorafenib in combination with RFA significantly improved the outcomes of early small HCC, which might be associated with decreased serum levels of active tumor growth factors HIF-1α. In addition, a combination of sorafenib and HIFs-targeted therapy or HIFs inhibitors can overcome HCC sorafenib resistance (Zeng et al., 2021). Besides, chloroquine significantly increased the apoptosis of HCC cells after IRFA and inhibited the enrichment of CSCs, and this effect was significantly enhanced by the combination of C-MET inhibitors (Zhao et al., 2018). Except for ablation, HIF inhibitors also play a promising role in treating early or unresectable HCC. For example, HIF inhibitor 32-134D was found to eradicate murine hepatocellular carcinoma in combination with anti-PD1 therapy (Salman et al., 2022). HIF-2α-targeted interventional chemoembolization was detected for the effective elimination of HCC (Chen S et al., 2022).

Taken together, RFA combined with other anti-cancer approaches (i.e. sorafenib, transarterial chemoembolization, and chloroquine) exerts a better curative effect in terms of tumor suppression than RFA alone, probably through inhibiting HIF-1α.

## Conclusion

HIF-1, 2α, the potent factors of tumor angiogenesis, play essential roles in the mechanisms of recurrence of HCC after ablation and are of great significance for the efficacy evaluation of ablation of patients with HCC and the development of individualized treatment options. HIF-1, 2α targets the different molecules (e.g., BNIP3, CA-IX, and arginase-1) and signaling cascades (e.g., VEGFA/EphA2 pathway) constituting a complex network that promotes HCC invasion and metastasis after ablation. Currently, the inhibitors of HIF have been developed, providing important proofof targeting HIF for the prevention of HCC recurrence after ablation. Though the effects of HIF-1, 2α on HCC after ablation have been preliminarily elucidated, the dynamic changes in HIF expression after ablation of HCC patients remain to be further studied.
